# Inflammatory-metabolic alterations in asthma-COPD overlap compared with COPD: evidence from NHANES and a hospital-based cohort

**DOI:** 10.3389/fmed.2026.1842221

**Published:** 2026-06-26

**Authors:** Xiaofei Yi, Yufen Fu, Yuxin Wang, Shiyue Li, Guoping Li

**Affiliations:** 1 State Key Laboratory of Respiratory Disease, Department of Clinical Laboratory, National Center for Respiratory Medicine, National Clinical Research Center for Respiratory Disease, Guangzhou Institute of Respiratory Health, The First Affiliated Hospital of Guangzhou Medical University, Guangzhou, Guangdong, China; 2 Laboratory of Allergy and Precision Medicine, Chengdu Institute of Respiratory Health, the Third People’s Hospital of Chengdu, Affiliated Hospital of Southwest Jiaotong University, Chengdu, China; 3 Department of Pulmonary and Critical Care Medicine, Chengdu Third People’s Hospital Branch of National Clinical Research Center for Respiratory Disease, Affiliated Hospital of ChongQing Medical University, Chengdu, China; 4 State Key Laboratory of Respiratory Disease, National Clinical Research Center for Respiratory Disease, Guangzhou Institute of Respiratory Health, The First Affiliated Hospital of Guangzhou Medical University, Guangzhou, China

**Keywords:** asthma, asthma-chronic obstructive pulmonary disease overlap, chronic obstructive pulmonary disease, inflammation, metabolism

## Abstract

**Background:**

Asthma-chronic obstructive pulmonary disease overlap (ACO) shares clinical characteristics with COPD but may be associated with a distinct inflammatory-metabolic profile. This study aimed to investigate inflammatory-metabolic biomarker profiles associated with ACO and to identify biomarkers that differentiate ACO from COPD.

**Methods:**

A two-cohort study was conducted. The National Health and Nutrition Examination Survey (NHANES) served as the primary cohort, while a hospital-based cohort from Chengdu was included as a complementary hospital-based clinical cohort. In NHANES, participants were classified into non-asthma/COPD, asthma-only, COPD-only and ACO groups. Weighted comparisons, pairwise analyses and multinomial logistic regression were performed to evaluate associations between 19 inflammatory-metabolic biomarkers and disease phenotypes. Variance inflation factor (VIF) analysis and least absolute shrinkage and selection operator (LASSO) regression were used to identify candidate biomarkers distinguishing ACO from COPD. Similar analyses were subsequently performed in the hospital-based cohort.

**Results:**

Participants with ACO exhibited a greater inflammatory-metabolic burden than individuals with asthma alone, COPD alone or neither condition. Pairwise comparisons showed that several metabolic biomarkers, particularly CTI, RCII, RFM, and LAP, remained significantly elevated in ACO compared with COPD alone. In multinomial logistic regression analyses, higher levels of inflammatory biomarkers (CLR, NLR, ELR, MLR, SII, and SIRI) and metabolic biomarkers (TyG and its derived indices, RFM, LAP, CTI, and RCII) remained significantly associated with ACO, whereas CALLY was inversely associated with ACO. In the NHANES cohort, LASSO regression identified ELR, MLR, CTI, and CALLY as the candidate biomarkers. The combined model (AUC = 0.580, 95% CI 0.550–0.611) showed the highest discriminatory performance among the evaluated biomarker combinations for distinguishing ACO from COPD. In the hospital-based cohort, LASSO regression selected TyG, AIP, CTI, MLR, and CLR. The combination of TyG, AIP, CTI, and MLR achieved the highest AUC (0.583, 95% CI 0.551–0.615).

**Conclusion:**

ACO was associated with inflammatory-metabolic alterations compared with COPD. Although the discriminatory performance of biomarker models was modest, findings from both cohorts consistently suggest an association between inflammatory-metabolic disturbances and the biological heterogeneity of ACO.

## 1 Introduction

Asthma-chronic obstructive pulmonary disease overlap (ACO) denotes a clinical situation wherein a patient displays characteristics of both asthma and chronic obstructive pulmonary disease (COPD) (1). The prevalence of ACO has been reported to range widely, from 1 to 30% in the general population to 10–60% in COPD and 15–66% in asthma (2–4). Compared with asthma or COPD alone, ACO is associated with a lower health-related quality of life, more frequent and severe acute exacerbations and a more rapid decline in lung function (5). Furthermore, ACO is associated with a higher medical burden, including more frequent hospitalizations and medical treatments (6–8). However, ACO remains a challenging and controversial clinical entity. The substantial overlap in symptoms, airflow limitation, inflammatory characteristics, disease progression, and smoking-related effects contributes to considerable heterogeneity across patients. Therefore, no universally accepted diagnostic criteria or individualized treatment strategies have yet been established.

Differentiation of ACO from COPD still represents an important clinical challenge. ACO has reversible airflow limitation and type 2 inflammation like asthma, but it also has irreversible airflow limitation and neutrophil-dominated Th1/Th17 immune responses like COPD (9, 10). Based on these characteristics, commonly used clinical indicators, such as bronchodilator response and blood eosinophil count, are unable to differentiate ACO from COPD. Previous studies have also focused on individual indicators to delineate the pathological features of ACO. For instance, elevated serum IgE and Fractional exhaled nitric oxide (FeNO) reflect Th2-polarized allergic inflammation and airway hyperresponsiveness, whereas biomarkers such as periostin, CXCL9, YKL-40, MCP-3 and neutrophil gelatinase-associated lipocalin (NGAL) have been proposed to differentiate ACO from asthma or COPD phenotypes (11–14). Recent studies suggest that eosinophil subtypes and chest CT characteristics may provide additional diagnostic information (7, 15), although their clinical utility remains uncertain. Beyond diagnostic uncertainty, ACO is a complex and heterogeneous disease characterized by chronic airway inflammation, immune dysregulation and metabolic disturbances (16–18). Compared with asthma or COPD, ACO is associated with more profound metabolic alterations (12, 19). However, these biomarkers reflect only a small portion of the disease biology and they may not fully represent the multifactorial nature of ACO. Therefore, integrative inflammatory-metabolic indices may provide a more comprehensive approach to characterizing ACO and distinguishing it from COPD.

Systemic inflammatory indices calculated from routine blood parameters, such as the CLR, NLR, PLR, MLR, ELR, SII, SIRI, and CALLY index, have demonstrated prognostic value in COPD, cardiovascular diseases and malignancies (20–22). In parallel, metabolic indices related to insulin resistance and adiposity, including the triglyceride-glucose (TyG) index and its derivatives, lipid accumulation product (LAP) and the C-reactive protein-triglyceride-glucose (CTI) index, have shown strong associations with cardiometabolic disorders (23–25). However, the potential roles of these inflammatory-metabolic indices in ACO have not been systematically investigated. In particular, it remains unclear whether specific inflammatory-metabolic patterns differentiate ACO from COPD. Whether ACO represents a unique biological phenotype or merely reflects the coexistence of asthma and COPD remains controversial. Identification of inflammatory-metabolic biomarkers may offer additional insight into the underlying mechanisms and biological heterogeneity of ACO.

In our study, we used nationally representative NHANES data as the primary cohort and a complementary hospital-based cohort as an independent clinical cohort. We compared inflammatory-metabolic biomarker profiles among individuals with non-asthma/COPD, asthma-only, COPD-only and ACO, and evaluated their associations with disease phenotypes using multinomial logistic regression analyses. We further applied variable selection approaches to identify candidate biomarkers that differentiate ACO from COPD, and examined whether similar biomarker patterns could be observed across population-based and clinical settings. Through these analyses, we aimed to identify inflammatory-metabolic biomarkers that differentiate ACO from COPD and to characterize the inflammatory-metabolic features associated with ACO.

## 2 Materials and methods

### 2.1 Study design and participants

#### 2.1.1 NHANES cohort

The primary analyses utilized data from the NHANES gathered from 2001 to 2018 and 2021–2023. Detailed NHANES data are publicly available at https://www.cdc.gov/nchs/nhanes/index.htm. A total of 103,284 participants were initially included. Participants were excluded if they were younger than 40 years, pregnant or had missing information required for the classification of asthma or COPD. After applying the eligibility criteria, 4,652 participants were included in the final analysis.

#### 2.1.2 Independent hospital-based cohort

An independent hospital-based cohort was established using electronic medical records from the Third People’s Hospital of Chengdu, Sichuan Province, China, between January 2013 and May 2023. A total of 10,307 patients with COPD were included, comprising 1,340 patients with ACO and 8,967 patients with COPD alone. Clinical and laboratory data were extracted from computerized medical records. For biomarkers with relatively stable biological variation (e.g., albumin, high-density lipoprotein cholesterol, low-density lipoprotein cholesterol, total cholesterol and blood cell count), patient-level mean values were calculated across available measurements. In contrast, biomarkers with rapid temporal variability (e.g., CRP, leukocyte subsets, fasting glucose and triglycerides) were represented by the first available measurement after hospital admission. Anthropometric variables, including height, weight and BMI were obtained from baseline records. Missing laboratory values were imputed using multiple imputation by chained equations with predictive mean matching (five imputations), with the exception of glucose, which was retained in its original form owing to its role in index calculation. Inflammatory and metabolic indices were subsequently derived using standardized formulas consistent with those applied in the NHANES cohort. Because waist circumference was not routinely recorded in the hospital-based cohort, waist-dependent indices including TyG-WC, TyG-WHtR, TyG-WWI, LAP, and RFM, could not be calculated and were therefore excluded from comparative analyses between cohorts.

**FIGURE 1:**
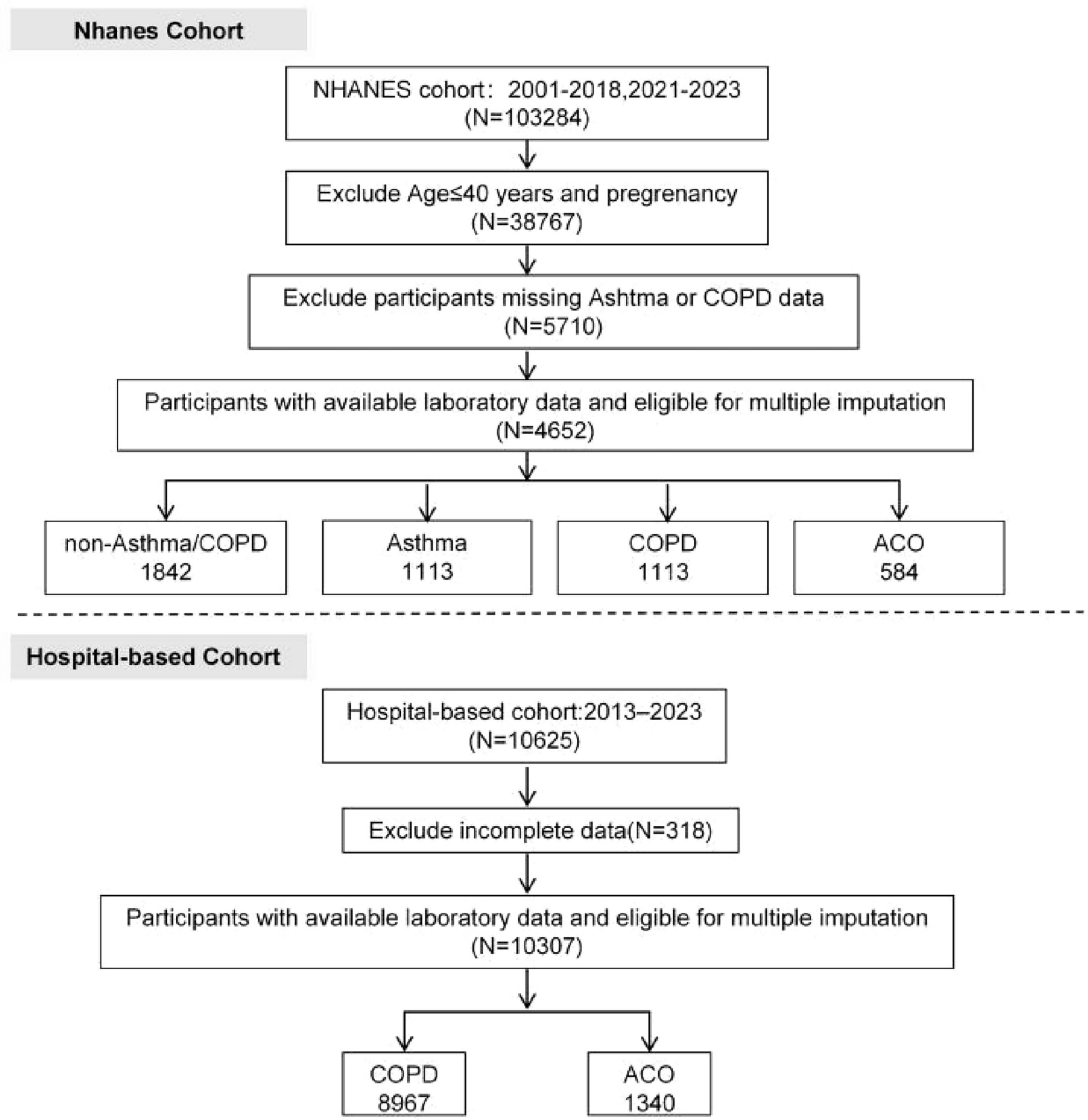
Study flowchart.

The detailed participant selection process is shown in Figure 1.

### 2.2 Definition of nineteen screening indicators and ACO

A total of 19 composite biomarkers were evaluated, as listed in Table 1, including nine inflammation-related indices (CLR, NLR, PLR, ELR, MLR, CALLY, SII, SIRI, and RCII) and ten metabolism-related indices (TyG, TyG-BMI, TyG-WC, TyG-WHtR, TyG-WWI, NHHR, RFM, LAP, AIP, and CTI). The corresponding indicators were collected from the NHANES database, including anthropometric indicators (height, weight, waist circumference) and laboratory indicators (blood cell count, fasting blood glucose, CRP, lipid-related indicators, etc.). Missing values in the analysis variables were imputed using multiple imputation by chained equations (MICE) implemented in the mice package in R. Subsequently, all indicators were calculated in accordance with their respective formulations.

**TABLE 1:** Formulas for the calculation of nineteen inflammatory-metabolic biomarkers.

Inflammation-related biomarkers	Metabolism-related biomarkers
CLR = CRP/lymphocyte	TyG = ln (TG × FPG/2)
NLR = neutrophil/lymphocyte	TyG-BMI = TyG × BMI
PLR = platelet/lymphocyte	TyG-WC = TyG × WC
ELR = eosinophil/lymphocyte	TyG-WHtR = TyG × WC/Height
MLR = monocyte/lymphocyte	TyG-WWI = TyG × WC/Weight
CALLY = albumin × lymphocyte/CRP	NHHR = (TC-HDL)/HDL
SII = platelet × neutrophil/ lymphocyte	RFM:Male = 64-(20 × Height/WC) Female = 76-(20 × Height/WC)
SIRI = monocyte × neutrophil/ lymphocyte	LAP:Male = (WC-65) × TG Female = (WC-58) × TG
RCII = (cholesterol-LDL-HDL) × CRP	AIP = log (TG/HDL)
	CTI = 0.412 × ln (CRP) + ln (TG × FPG/2)

For the NHANES cohort, people were diagnosed with COPD if they matched any of the following criteria: (1) a doctor told them they had COPD; (2) they had emphysema or chronic bronchitis in the past; (3) their forced expiratory volume in 1 s to forced vital capacity (FEV_1_/FVC) ratio was less than 0.7. Asthma was defined as: (1) a self-reported medical diagnosis of asthma; (2) a history of asthma episodes. Participants were classified as having ACO if they fulfilled the diagnostic criteria for both COPD and asthma (6, 10, 26, 27).

For the hospital-based cohort, COPD was diagnosed by respiratory specialists according to the GOLD criteria, based on compatible clinical symptoms and persistent airflow limitation on pulmonary function testing (post-bronchodilator FEV_1_/FVC < 0.70). Asthma was defined as a documented physician diagnosis of asthma, with disease onset before 40 years of age according to the medical records. Participants were classified as having ACO when they fulfilled the diagnostic criteria for both COPD and asthma.

### 2.3 Covariates

To examine the associations between the nineteen indices and ACO, potential confounding variables were chosen according to prior literature and clinical relevance. Covariates encompassed age, sex, race, BMI, marital status, educational status, smoking status, intake of alcohol and physical activity level. In addition, some comorbidities, including hypertension, heart failure, coronary heart disease, myocardial infarction, stroke, diabetes and cancer, were incorporated and classified as binary variables.

### 2.4 Statistical analysis

For NHANES analyses, sampling weights (WTSAF2YR), strata and primary sampling units were incorporated. This approach was based on the National Center for Health Statistics criteria for handling the complex multistage sample design and producing nationally representative results. Continuous variables were weighted means with standard errors (SE), while categorical variables were weighted proportions. Weighted linear regression and survey-weighted chi-square tests were used to compare baseline characteristics across the four disease groups (non-asthma/COPD, asthma-only, COPD-only and ACO). Pairwise comparisons among disease groups were performed using survey-weighted linear regression models. Because multiple pairwise comparisons were conducted within each biomarker, Bonferroni correction was applied to control the family-wise type I error rate.

Associations between inflammatory-metabolic biomarkers and disease phenotypes were evaluated using multinomial logistic regression analyses. Biomarkers were analyzed both as continuous variables and as quartiles. Three progressively adjusted models were constructed: the crude model; Model 1 adjusted for age, sex, race/ethnicity, education, marital status, and poverty-income ratio (PIR); Model 2 additionally adjusted for alcohol consumption, smoking status, and physical activity; and Model 3 further adjusted for major comorbidities. Given the large number of biomarkers evaluated, false discovery rate (FDR) correction using the Benjamini-Hochberg procedure was applied to the multinomial logistic regression analyses to account for multiple testing.

To identify candidate biomarkers associated with differences between ACO and COPD, variance inflation factor (VIF) analysis was first conducted to assess multicollinearity among candidate biomarkers. Biomarkers with VIF values < 10 were retained and subsequently entered into a least absolute shrinkage and selection operator (LASSO) logistic regression model with 10-fold cross-validation. Biomarkers retained by the LASSO model were further evaluated using logistic regression analyses comparing ACO and COPD.

Receiver operating characteristic (ROC) analyses were performed as exploratory analyses to assess the discriminatory ability of individual biomarkers and biomarker combinations for distinguishing ACO from COPD. Differences in the area under the curve (AUC) between competing models were evaluated using DeLong’s tests.

All statistical tests were two-sided, and *P* < 0.05 were considered statistically significant unless otherwise specified. All analyses were conducted using R software (version 4.5.0).

## 3 Results

### 3.1 Baseline characteristics of the participants

#### 3.1.1 NHANES cohort

After data screening and exclusion of participants with missing information, a total of 4,652 individuals were included and classified into four groups: non-asthma/COPD (*n* = 1,842), asthma alone (*n* = 1,113), COPD alone (*n* = 1,113) and ACO (*n* = 584). As demonstrated in Table 2, after applying NHANES sampling weights, significant differences were identified across nearly all baseline parameters among the four groups. Participants with ACO had a mean age of 59 ± 11 years and the highest BMI (31.05 ± 8.12 kg/m^2^). Females accounted for a greater proportion of the ACO group (65.0%) than the COPD-only group (49.9%). In terms of socioeconomic characteristics, individuals with ACO were more likely to be non-Hispanic White (75.5%), have lower income (15.5%), lower educational attainment and were more likely to be unmarried. Regarding lifestyle factors, current smoking was more prevalent in the ACO (30.9%) and COPD-alone (34.1%) groups compared with the non-asthma/COPD group (12.5%). Notably, the ACO group exhibited a substantially higher burden of comorbidities, including hypertension, heart failure, coronary heart disease, myocardial infarction, diabetes, stroke and cancer (all *P* < 0.001). Laboratory characteristics also differed significantly across groups. Compared with the other groups, participants with ACO showed higher levels of white blood cells, eosinophils, monocytes, neutrophils, platelet counts and C-reactive protein, together with lower serum albumin concentrations. Triglyceride levels were also highest in the ACO group. The distributions of inflammatory-metabolic composite biomarkers are presented in Table 3. Among the inflammatory indices, CLR, NLR, ELR, MLR, SII and SIRI were highest in the ACO group, whereas the CALLY index was markedly reduced (all *P* < 0.001). For metabolic indices, participants with ACO exhibited the highest levels of TyG, TyG-BMI, TyG-WC, TyG-WHtR, TyG-WWI, RFM, LAP, CTI and RCII (all *P* < 0.001). In contrast, PLR, AIP and NHHR did not differ significantly among groups. Overall, ACO exhibited a higher inflammatory burden and more severe metabolic abnormalities than those with asthma alone, COPD alone or no condition.

**TABLE 2:** Baseline demographic and clinical characteristics of participants in the NHANES cohort.

Characteristic	Non-asthma/COPD	ACO	Asthma_only	COPD_only	Overall	*P*-value^2^
Participants (million)	20.4^1^	6.8^1^	13.5^1^	13.1^1^	53.8^1^
Age	54 ± 10	59 ± 11	56 ± 11	61 ± 11	57 ± 11	< 0.001
BMI	29.3 ± 6.3	31.1 ± 8.1	31.0 ± 7.8	29.1 ± 7.1	29.9 ± 7.1	< 0.001
Gender						< 0.001
Male	882 (48.0%)	229 (35.0%)	435 (40.9%)	596 (50.1%)	2142 (45.1%)	
Female	960 (52.0%)	355 (65.0%)	678 (59.1%)	517 (49.9%)	2510 (54.9%)	
Education						0.004
< High school	259 (5.9%)	65 (5.8%)	106 (5.8%)	136 (6.2%)	566 (5.9%)	
Completed high school	276 (10.6%)	111 (15.3%)	129 (8.5%)	201 (13.9%)	717 (11.5%)	
> High school	1305 (83.6%)	408 (78.9%)	878 (85.7%)	775 (79.9%)	3366 (82.6%)	
Race						< 0.001
Mexican American	384 (8.0%)	30 (1.8%)	109 (4.3%)	86 (2.7%)	609 (5.0%)	
Other Hispanic	240 (4.9%)	61 (4.7%)	118 (6.3%)	72 (2.6%)	491 (4.7%)	
Non-Hispanic White	816 (71.8%)	341 (75.5%)	553 (69.1%)	729 (81.2%)	2439 (73.9%)	
Non-Hispanic Black	341 (10.5%)	112 (10.8%)	232 (12.6%)	171 (8.3%)	856 (10.6%)	
Other race	61 (4.8%)	40 (7.2%)	101 (7.6%)	55 (5.3%)	257 (5.9%)	
PIR						< 0.001
Poor	266 (8.3%)	126 (15.5%)	177 (11.3%)	189 (11.9%)	758 (10.9%)	
Not poor	1576 (91.7%)	458 (84.5%)	936 (88.7%)	924 (88.1%)	3894 (89.1%)	
Marital status						< 0.001
Married/living with a partner	1222 (72.6%)	297 (58.3%)	660 (68.5%)	645 (65.8%)	2824 (68.1%)	
Widowed/divorced/separated/ never married	620 (27.4%)	286 (41.7%)	452 (31.5%)	466 (34.2%)	1824 (31.9%)	
Alcohol	1226 (71.5%)	414 (72.7%)	779 (73.8%)	818 (78.2%)	3237 (73.8%)	0.046
Smoking status						< 0.001
Never	1032 (57.8%)	175 (31.2%)	594 (51.6%)	297 (27.9%)	2098 (45.6%)	
Former	522 (29.7%)	230 (37.8%)	358 (34.9%)	446 (38.0%)	1556 (34.1%)	
Current	287 (12.5%)	179 (30.9%)	158 (13.5%)	370 (34.1%)	994 (20.3%)	
Physical activity						< 0.001
Inactive	1,112 (58.6%)	242 (41.4%)	373 (33.4%)	482 (43.3%)	2,209 (46.9%)	
Moderate	411 (24.0%)	174 (37.8%)	335 (35.4%)	291 (32.6%)	1,211 (30.4%)	
Vigorous	67 (3.6%)	27 (5.3%)	43 (5.1%)	41 (4.9%)	178 (4.5%)	
Mixed intensity	252 (13.7%)	75 (15.5%)	248 (26.2%)	184 (19.1%)	759 (18.2%)	
HBP	876 (43.0%)	404 (64.1%)	648 (52.1%)	690 (54.5%)	2,618 (50.7%)	< 0.001
Heart failure	29 (1.2%)	92 (11.8%)	58 (4.2%)	110 (6.9%)	289 (4.7%)	< 0.001
Coronary heart disease	74 (3.4%)	75 (12.9%)	49 (3.8%)	140 (10.5%)	338 (6.4%)	< 0.001
Heart Attack	55 (2.6%)	93 (14.4%)	70 (4.7%)	148 (10.0%)	366 (6.4%)	< 0.001
Diabetes	331 (12.9%)	166 (21.9%)	285 (20.2%)	283 (20.1%)	1,065 (17.6%)	< 0.001
Stroke	53 (1.7%)	66 (10.6%)	72.0 (6.4%)	92 (6.0%)	283 (5.1%)	< 0.001
Cancer	153 (9.1%)	116 (22.5%)	173 (14.9%)	201 (18.2%)	643 (14.4%)	< 0.001
WBC (10^3^ cells/μL)	6.43 ± 2.27	7.57 ± 3.03	6.69 ± 1.97	7.15 ± 2.16	6.81 ± 2.32	< 0.001
Eos (10^3^ cells/μL)	0.19 ± 0.20	0.24 ± 0.18	0.24 ± 0.17	0.21 ± 0.13	0.21 ± 0.18	< 0.001
Lymphocyte (10^3^ cells/μL)	1.91 ± 0.68	2.16 ± 2.23	1.93 ± 0.68	1.95 ± 0.73	1.96 ± 1.02	0.068
Monocyte (10^3^ cells/μL)	0.51 ± 0.17	0.59 ± 0.21	0.52 ± 0.17	0.58 ± 0.20	0.54 ± 0.18	< 0.001
NEUT (10^3^ cells/μL)	3.77 ± 1.82	4.52 ± 1.78	3.96 ± 1.56	4.36 ± 1.78	4.06 ± 1.77	< 0.001
PLT (10^3^ cells/μL)	250.14 ± 68.06	261.42 ± 73.05	255.10 ± 64.96	251.89 ± 71.74	253.23 ± 68.94	0.020
CRP (mg/L)	3.63 ± 6.62	7.10 ± 11.89	4.75 ± 6.98	5.51 ± 12.40	4.81 ± 9.24	< 0.001
Albumin (g/L)	42.32 ± 2.94	40.66 ± 3.44	41.15 ± 3.26	41.22 ± 3.32	41.55 ± 3.24	< 0.001
Triglyceride (mmol/L)	1.37 ± 0.75	1.61 ± 1.22	1.47 ± 0.80	1.46 ± 0.70	1.45 ± 0.83	< 0.001
Cholesterol (mmol/L)	5.23 ± 1.01	5.17 ± 1.18	5.18 ± 1.03	5.04 ± 1.11	5.16 ± 1.07	< 0.001
HDL (mmol/L)	1.44 ± 0.42	1.46 ± 0.59	1.44 ± 0.42	1.38 ± 0.40	1.43 ± 0.44	0.114
LDL (mmol/L)	3.12 ± 0.88	2.97 ± 1.02	3.06 ± 0.91	2.98 ± 0.97	3.05 ± 0.93	0.002

^1^Mean ± SD; n (unweighted) (%).

^2^Pearson’s X^2^: Rao and Scott adjustment *P*-values were obtained from survey-weighted linear regression models for continuous variables and Rao–Scott χ^2^-tests for categorical variables.

**TABLE 3:** Distribution of inflammatory-metabolic biomarkers in the NHANES cohort.

Biomarker	Non-asthma-COPD	ACO	Asthma_only	COPD_only	Overall	*P*-value^2^
CLR	1.98 ± 4.03	4.26 ± 9.71	2.64 ± 4.78	3.24 ± 8.61	2.74 ± 6.51	< 0.001
NLR	2.12 ± 1.09	2.48 ± 1.50	2.21 ± 1.03	2.46 ± 1.28	2.27 ± 1.19	< 0.001
PLR	141.49 ± 53.53	142.03 ± 61.52	144.21 ± 54.47	142.53 ± 60.96	142.50 ± 56.68	0.307
ELR	0.11 ± 0.10	0.13 ± 0.12	0.13 ± 0.10	0.12 ± 0.08	0.12 ± 0.10	< 0.001
MLR	0.28 ± 0.11	0.32 ± 0.16	0.29 ± 0.13	0.32 ± 0.15	0.30 ± 0.13	< 0.001
CALLY	94.93 ± 150.33	52.88 ± 95.65	63.92 ± 102.65	74.46 ± 142.76	76.86 ± 132.54	< 0.001
SII	536.56 ± 491.61	647.50 ± 419.23	567.25 ± 322.64	622.10 ± 395.01	579.06 ± 423.74	< 0.001
SIRI	1.09 ± 0.72	1.53 ± 1.20	1.19 ± 0.78	1.47 ± 1.08	1.26 ± 0.92	< 0.001
TyG	1.25 ± 0.59	1.43 ± 0.65	1.35 ± 0.61	1.38 ± 0.55	1.33 ± 0.60	< 0.001
TyG-BMI	37.75 ± 20.98	45.80 ± 26.77	43.23 ± 24.53	41.40 ± 21.59	41.02 ± 23.01	< 0.001
TyG-WC	128.70 ± 67.72	151.28 ± 78.47	144.13 ± 75.57	144.71 ± 68.90	139.20 ± 71.90	< 0.001
TyG-WHtR	0.76 ± 0.40	0.91 ± 0.48	0.86 ± 0.45	0.86 ± 0.41	0.83 ± 0.43	< 0.001
TyG-WWI	13.94 ± 6.81	16.24 ± 7.69	15.34 ± 7.27	15.74 ± 6.64	15.01 ± 7.05	< 0.001
RFM	35.87 ± 7.74	39.35 ± 7.59	38.10 ± 8.29	36.08 ± 8.27	36.91 ± 8.10	< 0.001
LAP	56.10 ± 41.02	73.85 ± 66.05	66.97 ± 49.55	62.30 ± 43.17	62.50 ± 47.78	< 0.001
AIP	−0.14 ± 0.70	−0.01 ± 0.76	−0.07 ± 0.68	−0.01 ± 0.64	−0.07 ± 0.69	0.007
NHHR	2.88 ± 1.14	2.88 ± 1.38	2.85 ± 1.19	2.88 ± 1.26	2.87 ± 1.21	0.624
CTI	4.92 ± 9.80	10.85 ± 17.76	6.77 ± 10.76	7.49 ± 16.74	6.75 ± 13.30	< 0.001
RCII	2.54 ± 4.94	5.37 ± 8.58	3.35 ± 5.56	3.60 ± 7.32	3.36 ± 6.33	< 0.001

^1^Values are presented as weighted mean ± SD or unweighted n (%).

^2^*P*-values were obtained from survey-weighted linear regression models for continuous variables and Rao–Scott χ^2^-tests for categorical variables.

#### 3.1.2 Hospital-based cohort

The baseline characteristics of individuals with COPD and ACO in the hospital-based cohort are presented in Table 4. ACO patients were younger and more likely to be female than COPD patients, although their BMI and alcohol intake were similar. Patients with COPD exhibited a higher prevalence of cardiovascular comorbidities, including coronary heart disease and heart failure, whereas lung cancer was less frequent in the ACO group. In addition, respiratory failure was more prevalent among individuals with ACO, whereas in-hospital mortality was lower than that observed in patients with COPD. Several laboratory parameters differed significantly between groups. Compared with patients with COPD alone, individuals with ACO had higher levels of albumin, HDL-C, LDL-C, total cholesterol, eosinophils, neutrophils and white blood cells, whereas CRP levels were lower. Triglyceride levels and platelet counts were comparable between the two groups. The distributions of inflammatory-metabolic composite biomarkers are shown in Table 5. Among the inflammatory indices, PLR and MLR were lower in the ACO group, whereas the CALLY index was higher (all *P* < 0.001). No significant differences were observed for NLR, ELR, SII, or SIRI. Among the metabolic indices, AIP, CTI and NHHR were lower in the ACO group than in the COPD group (all *P* < 0.01), whereas TyG and TyG-BMI did not differ significantly between groups. Overall, demographic characteristics, comorbidity profiles and inflammatory-metabolic biomarker patterns differed between ACO and COPD in the hospital-based cohort.

**TABLE 4:** Baseline clinical characteristics of the hospital-based cohort.

Characteristic	COPD *N* = 8,967^1^	ACO *N* = 1,340^1^	*P*-value^2^
Age	78 ± 10	73 ± 10	< 0.001
BMI	22.1 ± 4.1	22.2 ± 4.0	0.4
Gender			< 0.001
Male	6,468 (72%)	796 (59%)	
Female	2,499 (28%)	544 (41%)	
Alcohol			0.6
Yes	281 (3.1%)	46 (3.4%)	
No	8686 (97%)	1294 (97%)	
Smoking status			0.047
Yes	697 (7.8%)	83 (6.2%)	
No	8270 (92%)	1257 (94%)	
HBP			0.13
Yes	5265 (59%)	757 (56%)	
No	3702 (41%)	583 (44%)	
Diabetes			0.4
Yes	2437 (27%)	349 (26%)	
No	6530 (73%)	991 (74%)	
Coronary heart disease			< 0.001
Yes	2127 (24%)	206 (15%)	
No	6840 (76%)	1134 (85%)	
Heart failure			< 0.001
Yes	795 (8.9%)	79 (5.9%)	
No	8172 (91%)	1261 (94%)	
Respiratory failure			0.026
Yes	2225 (25%)	371 (28%)	
No	6742 (75%)	969 (72%)	
Lung cancer			< 0.001
Yes	453 (5.1%)	27 (2.0%)	
No	8514 (95%)	1313 (98%)	
In-hospital mortality			< 0.001
No	8616 (96%)	1313 (98%)	
Yes	351 (3.9%)	27 (2.0%)	
Total cost (RMB)	20209 ± 25,673	18528 ± 19,779	0.005
Albumin (g/L)	35.5 ± 4.6	36.8 ± 4.1	< 0.001
HDL (mmol/L)	1.30 ± 0.39	1.42 ± 0.41	< 0.001
LDL (mmol/L)	2.38 ± 0.82	2.56 ± 0.84	< 0.001
Cholesterol (mmol/L)	4.13 ± 1.17	4.46 ± 1.18	< 0.001
CRP (mg/L)	30 ± 46	23 ± 40	< 0.001
Eos (1,000 cells/μL)	0.14 ± 0.20	0.17 ± 0.39	0.009
Lymphocyte (1,000 cells/μL)	1.25 ± 2.80	1.25 ± 0.63	> 0.9
NEUT (1,000 cells/μL)	5.7 ± 3.5	6.2 ± 3.5	< 0.001
WBC (1,000 cells/μL)	7.6 ± 4.5	8.1 ± 3.6	< 0.001
PLT (1,000 cells/μL)	178 ± 75	180 ± 61	0.2
Triglyceride (mmol/L)	1.15 ± 0.82	1.13 ± 0.74	0.3

^1^n (%); Mean ± SD.

^2^Pearson’s Chi-squared test; Welch Two Sample *t*-test.

**TABLE 5:** Inflammatory-metabolic biomarkers in the hospital-based cohort.

Biomarker	COPD *N* = 8,967^1^	ACO *N* = 1,340^1^	*P*-value^2^
NLR	7.2 ± 9.1	7.0 ± 8.2	0.5
PLR	202 ± 168	186 ± 133	< 0.001
ELR	0.13 ± 0.28	0.14 ± 0.34	0.5
MLR	0.58 ± 0.66	0.53 ± 0.47	< 0.001
SII	1,299 ± 1,863	1,277 ± 1,582	0.6
SIRI	67 ± 2.14	67 ± 1.30	> 0.9
CALLY	20 ± 4.8	25 ± 3.3	< 0.001
TyG	1.17 ± 0.61	1.17 ± 0.63	0.9
TyG-BMI	27 ± 1.6	27 ± 1.6	0.6
AIP	−0.21 ± 0.60	−0.32 ± 0.60	< 0.001
CTI	2.05 ± 0.92	1.90 ± 0.90	0.004
NHHR	0.00 ± 1.14	−0.11 ± 0.74	< 0.001

^1^n (%); Mean ± SD.

^2^Pearson’s Chi-squared test; Welch Two Sample *t*-test.

### 3.2 Differential inflammatory-metabolic profiles across disease groups

To further characterize differences among disease phenotypes, pairwise comparisons were performed (Table 6). When ACO was directly compared with COPD-only group, several metabolic biomarkers remained significantly elevated in the ACO group after FDR correction for multiple comparisons, including RFM, LAP, CTI and RCII (all adjusted *P* < 0.05). In contrast, most inflammatory biomarkers, including CLR, NLR, PLR, ELR, MLR, SII and SIRI, did not differ significantly between ACO and COPD-only participants after correction for multiple testing. Similarly, TyG and most TyG-derived indices showed no significant differences between the two groups.

**TABLE 6:** Pairwise comparisons of inflammatory and metabolic biomarkers among disease groups in the NHANES cohort.

Biomarker	Difference (β)	SE	Adjusted *P*-value
CLR	1.03	0.50	0.128
NLR	0.02	0.08	1.000
PLR	−0.50	4.15	1.000
ELR	0.01	0.01	0.147
MLR	0.00	0.01	1.000
CALLY	−21.58	8.99	0.054
SII	25.39	23.80	0.865
SIRI	0.06	0.06	1.000
TyG	0.05	0.04	0.904
TyG-BMI	4.40	1.88	0.064
TyG-WC	6.58	5.19	0.624
TyG-WHtR	0.05	0.03	0.278
TyG-WWI	0.50	0.51	0.984
RFM	3.27	0.52	< 0.001
LAP	11.55	4.31	0.025
AIP	0.01	0.05	1.000
NHHR	0.00	0.08	1.000
CTI	3.36	1.07	0.006
RCII	1.76	0.55	0.005

### 3.3 Logistic regression analyses

Multinomial logistic regression analyses were performed to further compare inflammatory-metabolic biomarkers between ACO and COPD-only participants in the NHANES cohort. As shown in Table 7, after adjustment for demographic characteristics, socioeconomic factors, lifestyle factors and comorbidities (Model 3), most inflammatory-metabolic biomarkers remained significantly associated with ACO after FDR correction. Among inflammatory biomarkers, higher levels of CLR, NLR, ELR, MLR, SII and SIRI were associated with increased odds of ACO, whereas CALLY was inversely associated with ACO. Among metabolic biomarkers, TyG and its derived indices, RFM, LAP, CTI, and RCII were positively associated with ACO. In contrast, AIP and NHHR showed inverse associations in the fully adjusted model.

**TABLE 7:** Multinomial logistic regression analyses of inflammatory-metabolic biomarkers associated with ACO compared with COPD-only participants in the NHANES cohort.

Biomarker	Crude model		Model 1		Model 2		Model 3	
	95% CI	FDR-P	95% CI	FDR-P	95% CI	FDR-P	95% CI	FDR-P
CLR	1.112 (1.005–1.231)	< 0.001	1.118 (1.117–1.119)	< 0.001	1.186 (1.185–1.187)	< 0.001	1.163 (1.161–1.164)	< 0.001
NLR	1.015 (1.014–1.016)	< 0.001	1.070 (1.069–1.071)	< 0.001	1.116 (1.115–1.117)	< 0.001	1.096 (1.094–1.097)	< 0.001
ELR	1.170 (1.168–1.171)	< 0.001	1.295 (1.294–1.297)	< 0.001	1.306 (1.304–1.307)	< 0.001	1.312 (1.311–1.314)	< 0.001
PLR	0.991 (0.990–0.992)	< 0.001	0.975 (0.974–0.976)	< 0.001	1.022 (1.021–1.023)	< 0.001	1.026 (1.025–1.027)	< 0.001
MLR	0.979 (0.979–0.980)	< 0.001	1.109 (1.108–1.110)	< 0.001	1.151 (1.150–1.152)	< 0.001	1.133 (1.132–1.134)	< 0.001
CALLY	0.811 (0.810–0.812)	< 0.001	0.808 (0.807–0.809)	< 0.001	0.766 (0.765–0.767)	< 0.001	0.778 (0.777–0.779)	< 0.001
SII	1.061 (1.060–1.062)	< 0.001	1.053 (1.052–1.054)	< 0.001	1.132 (1.131–1.133)	< 0.001	1.115 (1.114–1.116)	< 0.001
SIRI	1.052 (1.051–1.053)	< 0.001	1.137 (1.136–1.138)	< 0.001	1.192 (1.191–1.193)	< 0.001	1.165 (1.164–1.166)	< 0.001
TyG	1.079 (1.078–1.080)	< 0.001	1.112 (1.111–1.113)	< 0.001	1.088 (1.087–1.089)	< 0.001	1.068 (1.067–1.069)	< 0.001
TyG-BMI	1.195 (1.194–1.196)	< 0.001	1.207 (1.206–1.208)	< 0.001	1.199 (1.198–1.201)	< 0.001	1.187 (1.186–1.189)	< 0.001
TyG-WC	1.091 (1.090–1.092)	< 0.001	1.144 (1.143–1.146)	< 0.001	1.128 (1.127–1.130)	< 0.001	1.109 (1.108–1.111)	< 0.001
TyG-WHtR	1.125 (1.124–1.126)	< 0.001	1.154 (1.153–1.155)	< 0.001	1.140 (1.139–1.141)	< 0.001	1.119 (1.118–1.121)	< 0.001
TyG-WWI	1.071 (1.070–1.072)	< 0.001	1.106 (1.105–1.107)	< 0.001	1.081 (1.080–1.082)	< 0.001	1.057 (1.056–1.058)	< 0.001
RFM	1.509 (1.508–1.511)	< 0.001	1.519 (1.517–1.522)	< 0.001	1.513 (1.510–1.515)	< 0.001	1.494 (1.492–1.497)	< 0.001
LAP	1.233 (1.232–1.234)	< 0.001	1.243 (1.242–1.244)	< 0.001	1.239 (1.238–1.240)	< 0.001	1.226 (1.225–1.227)	< 0.001
AIP	1.012 (1.011–1.012)	< 0.001	1.048 (1.047–1.049)	< 0.001	1.011 (1.010–1.012)	< 0.001	0.986 (0.985–0.987)	< 0.001
NHHR	1.000 (0.999–1.001)	0.811	1.012 (1.011–1.013)	< 0.001	0.965 (0.964–0.966)	< 0.001	0.964 (0.963–0.965)	< 0.001
CTI	1.199 (1.198–1.200)	< 0.001	1.182 (1.181–1.183)	< 0.001	1.220 (1.219–1.221)	< 0.001	1.209 (1.208–1.210)	< 0.001
RCII	1.222 (1.221–1.223)	< 0.001	1.196 (1.195–1.197)	< 0.001	1.236 (1.235–1.237)	< 0.001	1.229 (1.228–1.230)	< 0.001

### 3.4 Biomarker selection and discrimination analyses in the NHANES cohort

**FIGURE 2:**
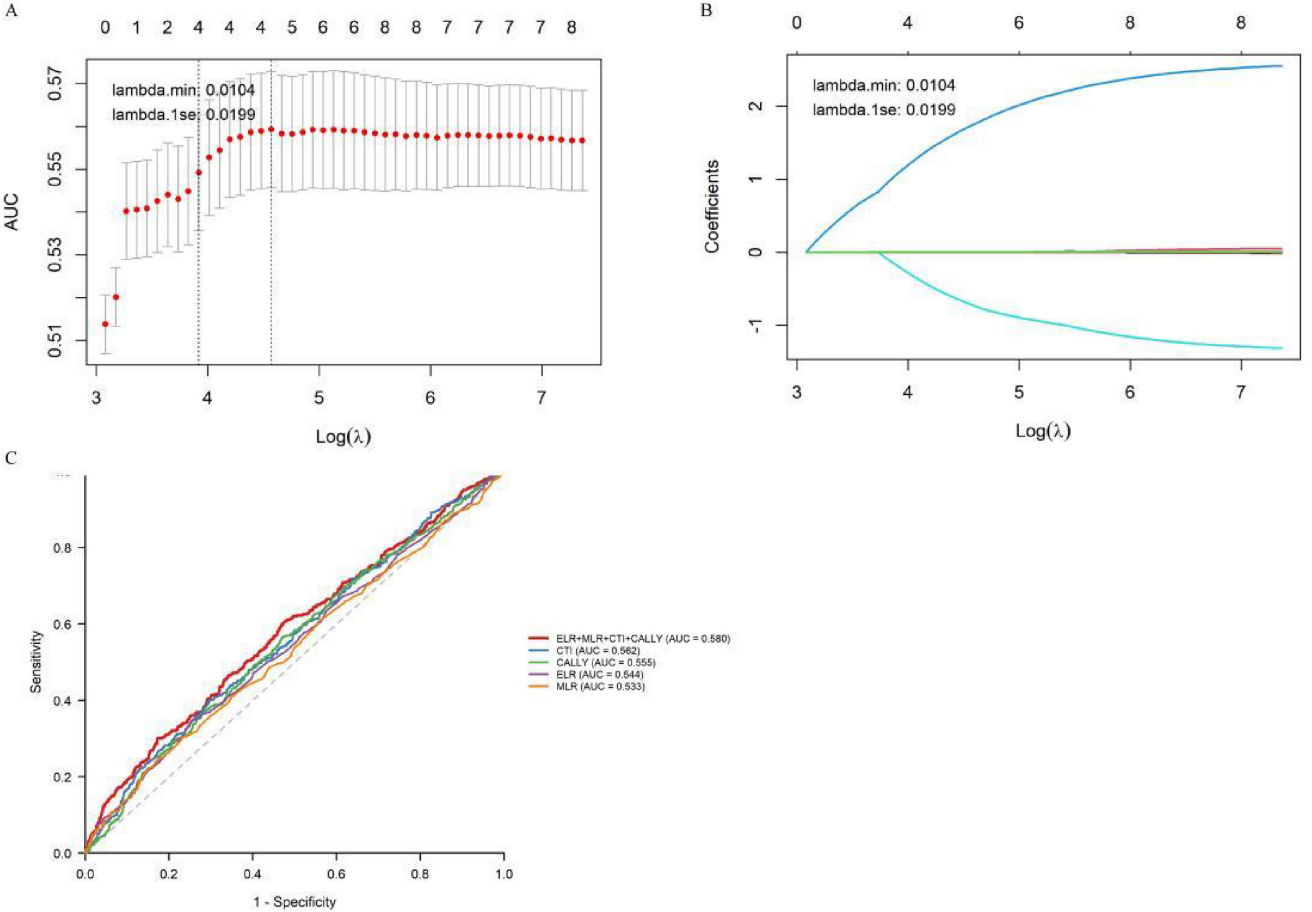
LASSO biomarker selection and ROC analysis in the NHANES cohort. **(A)** Ten-fold cross-validation curve of the LASSO logistic regression model. **(B)** Coefficient profiles of candidate inflammatory-metabolic biomarkers. **(C)** ROC curves of the selected biomarkers and the combined model.

After assessment of multicollinearity, biomarkers with VIF values ≥ 10 were excluded from further analyses (Supplementary Table 5). The remaining biomarkers were entered into a LASSO logistic regression model with 10-fold cross-validation. Using the lambda. Min criterion (λ = 0.0104), four biomarkers were retained, including ELR, MLR, CTI and CALLY (Supplementary Table 6 and Figures 2A,B). Among these variables, ELR had the largest absolute coefficient among the variables retained by the LASSO model (coefficient = 1.741), whereas MLR and CALLY were assigned negative coefficients. Among all evaluated combinations (Figure 2C), the combination of ELR, MLR, CTI and CALLY achieved the highest discriminatory performance for distinguishing ACO from COPD (AUC = 0.580, 95% CI 0.550–0.611). DeLong tests (Supplementary Table 7) showed that the combined model performed significantly better than ELR alone (AUC = 0.544, *P* = 0.014) and MLR alone (AUC = 0.533, *P* = 0.010). However, there were no significant differences when compared with CTI (AUC = 0.562, *P* = 0.265) or CALLY (AUC = 0.555, *P* = 0.151). Although the combined biomarker model showed modest improvement, the overall discriminative performance remained limited (AUC = 0.580).

### 3.5 Biomarker selection and discrimination analyses in the hospital-based cohort

**FIGURE 3:**
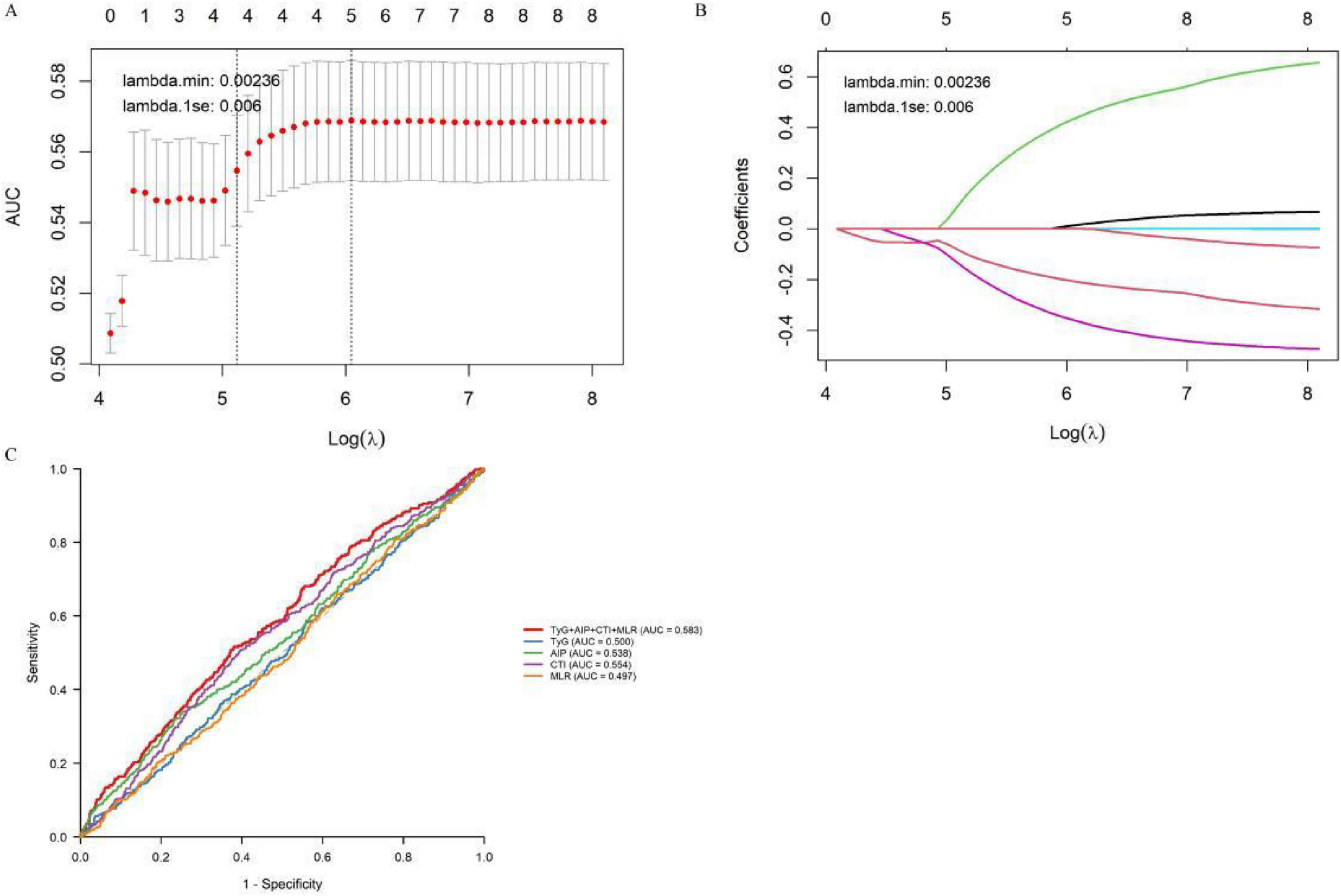
LASSO biomarker selection and ROC analysis in the hospital-based cohort. **(A)** Ten-fold cross-validation curve of the LASSO logistic regression model. **(B)** Coefficient profiles of candidate inflammatory-metabolic biomarkers. **(C)** ROC curves of the selected biomarkers and the combined model.

After excluding biomarkers with substantial multicollinearity (VIF ≥ 10), LASSO regression identified five biomarkers associated with differentiation between ACO and COPD in the hospital-based cohort, including TyG, AIP, CTI, MLR, and CLR (Supplementary Tables 8, 9; Figures 3A,B). Among these variables, TyG had the largest positive coefficient, whereas AIP, CTI and CLR were assigned negative coefficients in the LASSO model. Multivariable logistic regression further demonstrated that TyG was significantly associated with higher odds of ACO (OR = 1.905, 95% CI 1.364–2.660, *P* < 0.001), whereas AIP (OR = 0.607, 95% CI 0.459–0.802, *P* < 0.001) and CTI (OR = 0.754, 95% CI 0.609–0.932, *P* = 0.009) were inversely associated with ACO (Supplementary Table 11). In contrast, MLR and CLR were not significantly associated with ACO after multivariable adjustment. ROC analyses (Figure 3C) showed that the combination of TyG, AIP, CTI and MLR achieved the highest discriminative performance among the evaluated biomarker models for distinguishing ACO from COPD (AUC = 0.583, 95% CI 0.551–0.615). DeLong tests (Supplementary Table 10) indicated that the combined model achieved significantly higher AUC values than MLR alone (AUC = 0.497, *P* < 0.001), TyG alone (AUC = 0.500, *P* < 0.001), and AIP alone (AUC = 0.538, *P* = 0.009), whereas no significant difference was observed compared with CTI alone (AUC = 0.554, *P* = 0.068). Similar to the NHANES cohort, the combined inflammatory-metabolic biomarker model provided modest discrimination between ACO and COPD.

## 4 Discussion

In this study, we performed a comprehensive evaluation of inflammatory-metabolic biomarkers associated with ACO in a nationally representative NHANES cohort and a complementary hospital-based cohort. Several important findings emerged. First, individuals with ACO exhibited a greater inflammatory burden and more pronounced metabolic abnormalities than those with asthma alone, COPD alone or neither condition. Second, metabolic biomarkers, particularly CTI, RCII, RFM, and LAP, were more effective than most inflammatory markers in distinguishing ACO from COPD. Third, LASSO regression identified ELR, MLR, CTI, and CALLY as candidate biomarkers associated with differentiation between ACO and COPD. Finally, although combining these biomarkers modestly improved discrimination, the overall predictive performance remained limited.

The definition of ACO remains controversial and continues to evolve (28). Major international guidelines provide different perspectives on this condition. ACO was defined as concurrent COPD with self-reported, physician-diagnosed asthma before the age of 40 years. The 2025 GOLD report does not recognize ACO as a distinct disease entity but rather views it as the coexistence of asthma and COPD with overlapping treatable traits (29). In contrast, the 2024 GINA report classified ACO as a kind of clinical overlap syndrome, whose prognosis is worse than that of asthma only or COPD only (30). Against this background, the identification of objective biomarkers may provide additional insight into the biological characteristics of ACO beyond conventional clinical definitions. Pairwise comparisons demonstrated that several metabolic biomarkers, particularly CTI, RCII, RFM, and LAP, remained significantly elevated in ACO compared with COPD alone. Moreover, multinomial logistic regression allowed direct comparisons between ACO, COPD-only and asthma-only groups, thereby reducing the potential bias introduced by treating ACO as a simple binary outcome. Collectively, these findings suggest that ACO is associated with inflammatory-metabolic characteristics that differ from those observed in COPD alone. However, the present cross-sectional study was unable to determine whether these differences reflect a distinct biological process or the combined effects of asthma and COPD.

Notably, biomarkers selected by LASSO were not completely identical to those identified in pairwise comparisons. This discrepancy is expected, because pairwise analyses assess differences in single biomarkers, while LASSO selects variables that offer additional information for distinguishing ACO from COPD in a multivariable context. Therefore, inflammatory biomarkers such as ELR and MLR were kept, although their univariable differences were small, suggesting they may provide additional information when used together with metabolic markers. Consistent with this observation, the optimal model incorporated both inflammatory and metabolic biomarkers, suggesting that inflammatory and metabolic alterations may provide complementary information regarding the heterogeneity associated with ACO.

Compared with the NHANES cohort, several inflammatory-metabolic biomarkers showed different patterns in the hospital-based cohort. For example, CTI was positively associated with ACO in NHANES but inversely associated with ACO in the hospital-based cohort. These discrepancies may reflect substantial differences in study populations and clinical settings. The NHANES cohort is composed of community individuals, among which includes stable patients with COPD, asthma, and ACO. However, the hospital cohort consists of ACO and COPD patients who were admitted because of acute exacerbation. Biomarker measurements obtained during hospitalization may also have been influenced by acute clinical status and ongoing treatment (31, 32). Furthermore, the LASSO regression detected different biomarkers in the two cohorts, indicating that the relative contribution of inflammatory-metabolic pathways may differ between populations. Nevertheless, both cohorts support the involvement of inflammatory-metabolic disturbances in ACO and highlight the heterogeneity of this condition.

Participants with ACO differed from the other groups in age, BMI, smoking exposure, socioeconomic status, and comorbidity burden, all of which may influence inflammatory and metabolic biomarkers. Consistent with the findings of Zhu et al. (6), patients with ACO in the NHANES cohort had a substantial burden of comorbidities, characterized by a markedly elevated prevalence of hypertension, heart failure, coronary heart disease, myocardial infarction, diabetes, stroke and cancer. In contrast, ACO inpatients in the hospital-based cohort had a lower incidence of comorbidities compared to patients with COPD alone. This disparity may be due to changes in the type of patients and how they are admitted to the hospital. To reduce the impact of these potential confounders, we performed multinomial logistic regression analyses with progressive adjustment for demographic factors, lifestyle characteristics, socioeconomic variables, and major comorbidities. Notably, several inflammatory and metabolic biomarkers remained associated with ACO after full adjustment. Nevertheless, residual confounding cannot be completely excluded, and part of the observed biomarker heterogeneity may reflect differences in disease severity and clinical characteristics rather than ACO-specific biological mechanisms.

Our findings further support the involvement of inflammatory-metabolic disturbances in ACO. Several inflammatory biomarkers, including CLR, NLR, ELR, MLR, SII, and SIRI, remained significantly associated with ACO after multivariable adjustment. These observations are broadly consistent with previous studies suggesting that ACO is characterized by a more complex inflammatory profile than COPD alone. For example, Fangal et al. reported that ACO was associated with higher white blood cell and eosinophil counts than COPD, whereas neutrophil-related responses were less pronounced (5). In addition, lower CALLY levels were consistently associated with ACO. Because CALLY reflects inflammation, immune function and nutritional status, this finding is consistent with earlier data that hunger and chronic inflammation are strongly associated with worse respiratory outcomes (33–35). In addition to inflammation, metabolic alterations may also contribute to the biological heterogeneity of ACO (36). In the present study, pairwise comparisons demonstrated that CTI, RCII, RFM and LAP remained significantly elevated in ACO compared with COPD alone, while multinomial logistic regression further confirmed associations for multiple metabolic biomarkers. Although TyG itself did not show a marked difference in direct comparisons between ACO and COPD, TyG and several TyG-derived indices remained associated with ACO after multivariable adjustment. The TyG index and its related measures are widely accepted as reliable indicators of insulin resistance. Insulin resistance has been linked to oxidative stress, mitochondrial dysfunction and systemic inflammation (37), which are linked to an increased incidence, prevalence, and severity of COPD and asthma (38–40). The increase in LAP is closely related to the accumulation of visceral fat. Studies have shown that LAP is positively associated with metabolic disorders, and is considered to be an important metabolic driver of insulin resistance (41). CTI is a composite biomarker that integrates inflammatory and metabolic information. Emerging evidence has shown that CTI is closely related to a variety of chronic diseases, including depression, diabetes and coronary artery disease (42–44). These findings suggest that metabolic dysfunction and systemic inflammation may both contribute to the inflammatory-metabolic heterogeneity associated with ACO.

However, several limitations should be acknowledged. First, although false discovery rate correction was applied to the primary analyses and Bonferroni correction was used for pairwise comparisons, residual false-positive findings cannot be completely excluded because of the large number of statistical tests performed (45). Second, the present study included only participants from NHANES and a single hospital-based cohort. Future studies involving larger and more diverse populations from different geographic regions are needed to improve generalizability. Moreover, our findings should be interpreted in the context of larger and more deeply phenotyped ACO and COPD cohorts. Previous studies (5) from cohorts such as COPDGene, SPIROMICS, and ECLIPSE have substantially advanced the understanding of COPD heterogeneity and overlap phenotypes through detailed clinical characterization, pulmonary function testing, imaging assessments, longitudinal follow-up, and molecular profiling. In contrast, the present study relied primarily on routinely available blood-based inflammatory-metabolic biomarkers and therefore provided a lower-resolution assessment of disease heterogeneity. Third, important clinical variables relevant to ACO, including FeNO measurements and detailed pulmonary function parameters, were unavailable for all participants. Fourth, the discriminative performance of the biomarker models was modest in both cohorts. These findings indicate that inflammatory-metabolic biomarkers alone have limited value for distinguishing ACO from COPD in clinical practice. Therefore, the identified biomarkers should be regarded primarily as exploratory indicators of inflammatory-metabolic heterogeneity rather than clinically actionable diagnostic tools.

## 5 Conclusion

ACO was associated with inflammatory-metabolic alterations compared with COPD. Although the discriminatory performance of biomarker models was modest, findings from both cohorts consistently support that inflammatory-metabolic disturbances may contribute to the biological heterogeneity of ACO.

## Data Availability

The raw data supporting the conclusions of this article will be made available by the authors, without undue reservation.
