# Supplementary material for: Inflammatory-metabolic alterations in asthma-COPD overlap compared with COPD: evidence from NHANES and a hospital-based cohort
Source: Front Med (Lausanne). 2026 Jun 26;13:1842221. doi: 10.3389/fmed.2026.1842221 (PMC13350444; doi:10.3389/fmed.2026.1842221)
Supplement: Supplementary file 1 [file Data_Sheet_1.PDF]

Supplementary Table 1. Inflammatory-metabolic biomarkers among disease groups in the NHANES cohort

Biomarker	Asthma only<br>N = 13.5 million[1]	COPD only<br>N = 13.1 million[1]	ACO<br>N = 6.8 million[1]	p-value[2]
CLR	2.64 ± 4.78	3.24 ± 8.61	4.26 ± 9.71	*<0.001*
NLR	2.21 ± 1.03	2.46 ± 1.28	2.48 ± 1.50	*0.003*
PLR	144.21 ± 54.47	142.53 ± 60.96	142.03 ± 61.52	*0.170*
ELR	0.13 ± 0.10	0.12 ± 0.08	0.13 ± 0.12	*0.138*
MLR	0.29 ± 0.13	0.32 ± 0.15	0.32 ± 0.16	*0.005*
CALLY	63.92 ± 102.65	74.46 ± 142.76	52.88 ± 95.66	*<0.001*
SII	567.25 ± 322.65	622.10 ± 395.02	647.50 ± 419.25	*0.005*
SIRI	1.19 ± 0.78	1.47 ± 1.08	1.53 ± 1.20	*<0.001*
TyG	1.35 ± 0.61	1.38 ± 0.55	1.43 ± 0.65	*0.057*
TyG-BMI	43.23 ± 24.53	41.40 ± 21.59	45.80 ± 26.77	*0.058*
TyG-WC	144.13 ± 75.57	144.71 ± 68.90	151.28 ± 78.48	*0.177*
TyG-WHtR	0.86 ± 0.45	0.86 ± 0.41	0.91 ± 0.48	*0.115*
TyG-WWI	15.34 ± 7.27	15.74 ± 6.64	16.24 ± 7.69	*0.073*
RFM	38.10 ± 8.29	36.08 ± 8.27	39.35 ± 7.59	*<0.001*
LAP	66.97 ± 49.55	62.30 ± 43.17	73.85 ± 66.06	*0.016*
AIP	-0.07 ± 0.68	-0.01 ± 0.64	-0.01 ± 0.76	*0.208*
NHHR	2.85 ± 1.19	2.88 ± 1.26	2.88 ± 1.38	*0.852*
CTI	6.77 ± 10.76	7.49 ± 16.74	10.85 ± 17.76	*<0.001*
RCII	3.35 ± 5.56	3.60 ± 7.33	5.37 ± 8.58	*<0.001*

Supplementary Table 2. Pairwise comparisons of inflammatory and metabolic biomarkers among disease groups in the NHANES cohort

Biomarker	Comparison	Difference (β)	SE	Adjusted P value
CLR	ACO vs Asthma only	1.62	0.40	*<0.001*
	ACO vs COPD only	1.03	0.50	*0.128*
	Asthma only vs COPD only	-0.59	0.32	*0.196*
NLR	ACO vs Asthma only	0.27	0.07	*<0.001*
	ACO vs COPD only	0.02	0.08	*1.000*
	Asthma only vs COPD only	-0.25	0.06	*<0.001*
PLR	ACO vs Asthma only	-2.18	3.66	*1.000*
	ACO vs COPD only	-0.50	4.15	*1.000*
	Asthma only vs COPD only	1.68	3.49	*1.000*
ELR	ACO vs Asthma only	0.00	0.01	*1.000*

Biomarker	Comparison	Difference (β)	SE	Adjusted P value
	ACO vs COPD only	0.01	0.01	*0.147*
	Asthma only vs COPD only	0.01	0.01	*0.021*
	ACO vs Asthma only	0.03	0.01	*0.009*
MLR	ACO vs COPD only	0.00	0.01	*1.000*
	Asthma only vs COPD only	-0.03	0.01	*0.001*
	ACO vs Asthma only	-11.04	6.37	*0.257*
CALLY	ACO vs COPD only	-21.58	8.99	*0.054*
	Asthma only vs COPD only	-10.54	8.83	*0.706*
	ACO vs Asthma only	80.24	21.65	*<0.001*
SII	ACO vs COPD only	25.39	23.80	*0.865*
	Asthma only vs COPD only	54.85	18.19	*0.009*
	ACO vs Asthma only	0.34	0.06	*<0.001*
SIRI	ACO vs COPD only	0.06	0.06	*1.000*
	Asthma only vs COPD only	-0.28	0.05	*<0.001*
	ACO vs Asthma only	0.08	0.04	*0.133*
TyG	ACO vs COPD only	0.05	0.04	*0.904*
	Asthma only vs COPD only	-0.03	0.03	*1.000*
	ACO vs Asthma only	2.57	1.51	*0.272*
TyG-BMI	ACO vs COPD only	4.40	1.88	*0.064*
	Asthma only vs COPD only	1.83	1.42	*0.604*
	ACO vs Asthma only	7.15	4.85	*0.430*
TyG-WC	ACO vs COPD only	6.58	5.19	*0.624*
	Asthma only vs COPD only	-0.58	4.52	*1.000*
	ACO vs Asthma only	0.05	0.03	*0.340*
TyG-WHtR	ACO vs COPD only	0.05	0.03	*0.278*
	Asthma only vs COPD only	0.01	0.03	*1.000*
	ACO vs Asthma only	0.90	0.47	*0.183*
TyG-WWI	ACO vs COPD only	0.50	0.51	*0.984*
	Asthma only vs COPD only	-0.40	0.43	*1.000*
	ACO vs Asthma only	1.24	0.55	*0.075*
RFM	ACO vs COPD only	3.27	0.52	*<0.001*
	Asthma only vs COPD only	2.02	0.61	*0.004*
LAP	ACO vs Asthma only	6.88	4.13	*0.294*

Biomarker	Comparison	Difference (β)	SE	Adjusted P value
	ACO vs COPD only	11.55	4.31	*0.025*
	Asthma only vs COPD only	4.67	2.88	*0.323*
	ACO vs Asthma only	0.06	0.05	*0.573*
AIP	ACO vs COPD only	0.01	0.05	*1.000*
	Asthma only vs COPD only	-0.05	0.04	*0.601*
	ACO vs Asthma only	0.03	0.07	*1.000*
NHHR	ACO vs COPD only	0.00	0.08	*1.000*
	Asthma only vs COPD only	-0.03	0.08	*1.000*
	ACO vs Asthma only	4.08	0.86	*<0.001*
CTI	ACO vs COPD only	3.36	1.07	*0.006*
	Asthma only vs COPD only	-0.72	0.66	*0.839*
	ACO vs Asthma only	2.02	0.50	*<0.001*
RCII	ACO vs COPD only	1.76	0.55	*0.005*
	Asthma only vs COPD only	-0.26	0.31	*1.000*

Supplementary Table 4. Multinomial logistic regression analyses of inflammatory-metabolic biomarkers associated with ACO compared with Asthma-only participants in the NHANES cohort

Biomarkers	Crude model		Model 1		Model 2		Model 3	
	95%CI	FDR-P	95%CI	FDR-P	95%CI	FDR-P	95%CI	FDR-P
CLR	1.292 (1.291-1.294)	*<0.001*	1.250 (1.249-1.252)	*<0.001*	1.335 (1.333-1.337)	*<0.001*	1.334 (1.332-1.336)	*<0.001*
NLR	1.310 (1.308-1.311)	*<0.001*	1.259 (1.258-1.260)	*<0.001*	1.265 (1.263-1.266)	*<0.001*	1.246 (1.245-1.248)	*<0.001*
ELR	0.985 (0.984-0.986)	*<0.001*	0.972 (0.971-0.973)	*<0.001*	0.984 (0.983-0.985)	*<0.001*	0.979 (0.978-0.980)	*<0.001*
PLR	0.963 (0.962-0.963)	*<0.001*	0.951 (0.950-0.952)	*<0.001*	0.954 (0.953-0.955)	*<0.001*	0.937 (0.936-0.938)	*<0.001*
MLR	1.270 (1.269-1.272)	*<0.001*	1.228 (1.226-1.229)	*<0.001*	1.201 (1.199-1.202)	*<0.001*	1.172 (1.170-1.173)	*<0.001*
CALLY	0.873 (0.872-0.874)	*<0.001*	0.916 (0.915-0.917)	*<0.001*	0.888 (0.887-0.889)	*<0.001*	0.897 (0.896-0.898)	*<0.001*
SII	1.268 (1.267-1.269)	*<0.001*	1.240 (1.239-1.242)	*<0.001*	1.293 (1.292-1.295)	*<0.001*	1.274 (1.272-1.275)	*<0.001*
SIRI	1.556 (1.555-1.558)	*<0.001*	1.494 (1.492-1.496)	*<0.001*	1.498 (1.496-1.500)	*<0.001*	1.474 (1.472-1.476)	*<0.001*
TyG	1.140 (1.138-1.141)	*<0.001*	1.121 (1.120-1.122)	*<0.001*	1.111 (1.110-1.112)	*<0.001*	1.153 (1.152-1.154)	*<0.001*
TyG-BMI	1.105 (1.104-1.106)	*<0.001*	1.093 (1.092-1.094)	*<0.001*	1.102 (1.101-1.103)	*<0.001*	1.151 (1.149-1.152)	*<0.001*
TyG-WC	1.099 (1.098- 1.100)	*<0.001*	1.085 (1.084-1.086)	*<0.001*	1.085 (1.084-1.086)	*<0.001*	1.134 (1.133-1.135)	*<0.001*
TyG-WHtR	1.109 (1.108- 1.110)	*<0.001*	1.073 (1.072-1.074)	*<0.001*	1.075 (1.074-1.076)	*<0.001*	1.122 (1.120-1.123)	*<0.001*
TyG-WWI	1.133 (1.132-1.134)	*<0.001*	1.090 (1.089-1.091)	*<0.001*	1.080 (1.079-1.081)	*<0.001*	1.123 (1.122-1.125)	*<0.001*
RFM	1.172 (1.171-1.173)	*<0.001*	1.026 (1.024-1.028)	*<0.001*	1.022 (1.020-1.023)	*<0.001*	1.048 (1.047-1.050)	*<0.001*
LAP	1.115 (1.114-1.115)	*<0.001*	1.104 (1.103-1.105)	*<0.001*	1.125 (1.124-1.126)	*<0.001*	1.161 (1.160-1.162)	*<0.001*
AIP	1.092 (1.091-1.093)	*<0.001*	1.103 (1.102-1.104)	*<0.001*	1.104 (1.103-1.106)	*<0.001*	1.126 (1.125-1.127)	*<0.001*
NHHR	1.020 (1.019-1.021)	*<0.001*	1.069 (1.068-1.070)	*<0.001*	1.074 (1.073-1.075)	*<0.001*	1.094 (1.093-1.096)	*<0.001*
CTI	1.299 (1.297-1.300)	*<0.001*	1.274 (1.273-1.276)	*<0.001*	1.384 (1.383-1.386)	*<0.001*	1.433 (1.431-1.435)	*<0.001*
RCII	1.291 (1.289-1.292)	*<0.001*	1.276 (1.275-1.278)	*<0.001*	1.388 (1.386-1.389)	*<0.001*	1.429 (1.427-1.431)	*<0.001*

Supplementary Table 5.VIF analysis of candidate inflammatory-metabolic biomarkers in the NHANES cohort

Biomarker	VIF
CLR	3.663
NLR	5.740
PLR	6.626
ELR	1.256
MLR	8.313
CALLY	1.100
SII	13.878
SIRI	15.345
TyG	4.380
AIP	4.188
CTI	3.611

Supplementary Table 6. Biomarkers selected by LASSO regression for differentiating ACO from COPD in the NHANES cohort

Biomarker	Coefficient
ELR	1.741
MLR	-0.720
CTI	0.004
CALLY	-0.0004

Supplementary Table 7. DeLong comparisons of ROC curves between the optimal combined model and single-biomarker models in the NHANES cohort

Reference Model	Compared Model	AUC Reference	AUC Compared	DeLong Test P value
ELR+MLR+CTI+CALLY	MLR	0.580	0.533	*0.01*
ELR+MLR+CTI+CALLY	ELR	0.580	0.544	*0.014*
ELR+MLR+CTI+CALLY	CALLY	0.580	0.555	*0.151*
ELR+MLR+CTI+CALLY	CTI	0.580	0.562	*0.265*

Supplementary Table 8.VIF analysis of candidate inflammatory-metabolic biomarkers in the hospital-based cohort

Biomarker	VIF
SII	13.690
SIRI	13.134
NLR	11.572
PLR	7.460
CTI	6.287
TyG	4.952
CLR	2.536
CALLY	2.517
AIP	2.212
MLR	1.940
ELR	1.043

Supplementary Table 9. Biomarkers selected by LASSO regression for differentiating ACO from COPD in the hospital-based cohort

Biomarker	Coefficient
TyG	0.432
AIP	-0.359
CTI	-0.206
MLR	0.013
CLR	-0.0006

Supplementary Table 10. DeLong comparisons of ROC curves between the optimal combined model and single-biomarker models in the hospital-based cohort

Reference Model	Compared Model	AUC Reference	AUC Compared	DeLong Test P value
TyG+AIP+CTI+MLR	MLR	0.583	0.497	*<0.001*
TyG+AIP+CTI+MLR	TyG	0.583	0.500	*<0.001*
TyG+AIP+CTI+MLR	AIP	0.583	0.538	*0.009*
TyG+AIP+CTI+MLR	CTI	0.583	0.554	*0.068*

Supplementary Table 11. Logistic regression analyses of LASSO-selected biomarkers associated with ACO in the hospital-based cohort

Biomarker	Univariable OR (95% CI)	Univariable P value	Multivariable OR (95% CI)	Multivariable P value
TyG	1.013 (0.842–1.219)	*0.887*	1.905 (1.364–2.660)	*<0.001*
AIP	0.788 (0.648–0.958)	*0.017*	0.607 (0.459–0.802)	*<0.001*
CTI	0.832 (0.733–0.944)	*0.004*	0.754 (0.609–0.932)	*0.009*
MLR	0.970 (0.823–1.143)	*0.716*	1.071 (0.919–1.249)	*0.379*
CLR	0.998 (0.996–0.999)	*0.012*	0.999 (0.997–1.001)	*0.421*
